# Population-based study on birth outcomes among women with hypertensive disorders of pregnancy and gestational diabetes mellitus

**DOI:** 10.1038/s41598-021-96345-0

**Published:** 2021-08-30

**Authors:** Ya-Wen Lin, Ming-Hung Lin, Lee-Wen Pai, Jen-Wei Fang, Chih-Hsin Mou, Fung-Chang Sung, Ya-Ling Tzeng

**Affiliations:** 1 School of Nursing and Graduate Institute of Nursing, China Medical University, Shui-Nan Campus, 100 Jingmao Rd. Sec. 1, Taichung, 406040 Taiwan; 2 Department of Public Health, China Medical University, Taichung, Taiwan; 3 Department of Pharmacy and Master Program, Tajen University, Pingtung, Taiwan; 4 Department of Nursing, Central Taiwan University of Science and Technology, Taichung, Taiwan; 5 Department of Obstetrics and Gynecology, China Medical University Hospital, Taichung, Taiwan; 6 Management Office for Health Data, China Medical University Hospital, Taichung, Taiwan; 7 Department of Health Services Administration, China Medical University, Taichung, Taiwan; 8 Department of Food Nutrition and Health Biotechnology, Asia University, Taichung, Taiwan; 9 Department of Nursing, China Medical University Hospital, Taichung, Taiwan

**Keywords:** Hypertension, Gestational diabetes

## Abstract

To evaluate birth outcomes in women with hypertensive disorders of pregnancy (HDP) and gestational diabetes mellitus (GDM), we used insurance data of Taiwan to evaluate 11 adverse neonatal outcomes of infants born to women with HDP (N = 7775) and with both HDP and GDM (HDP/GDM) (N = 1946), comparing to women with neither disorder (N = 19,442), matched by age. The impacts of preeclampsia/eclampsia were also evaluated. Results showed that Caesarean section delivery was near 1.7-fold greater in the HDP/GDM and HDP groups than in comparisons. The preterm delivery rates were more than threefold greater in HDP/GDM group and HDP group than in comparisons with adjusted odds ratios (aORs) of 4.84 (95% confidence interval (CI) 4.34–5.40) and 3.92 (95% CI 3.65–4.21), respectively, followed by jaundice (aORs 2.95 (95% CI 2.63–3.33) and 1.90 (95% CI 1.76–2.06)), and small gestation age (SGA) (aORs 6.57 (95% CI 5.56–7.75) and 5.81 (95% CI 5.15–6.55)). Incidence rates of birth trauma, patent ductus arteriosus, atrial septal defect, respiratory distress syndrome, and neonatal hypoglycemia were also higher in the HDP/GDM and HDP groups than in the comparison group. Most adverse outcomes increased further in women with preeclampsia or eclampsia. In conclusion, women with HDP are at elevated risks of adverse neonatal outcomes. Risks of most adverse outcomes increase further for women with both HDP and GDM. Preeclampsia or eclampsia may also contribute to these outcomes to higher risk levels. Every pregnant woman with these conditions deserves specialized prenatal care.

## Introduction

Hypertensive disorders of pregnancy (HDP) and gestational diabetes mellitus (GDM) are common disorders that may contribute to complications in pregnant women and newborns. The prevalence of HDP ranges from 5 to 10%^1–3^. Approximately 8.7–14% of pregnant women develop GDM^4–6^. Both disorders are important global public health concerns.

A WHO systemic analysis showed that hypertensive disorders accounted for 14.0% of maternal deaths in 2003–2009^7^. Women with gestational hypertension (GHT) may progress to preeclampsia and eclampsia with proteinuria, edema, and tonic–clonic seizures after 20 weeks of gestation. These conditions can trigger acute liver rupture, chronic kidney disease, visual loss, and other maternal complications^8–12^. It can also pose a higher risk for adverse birth outcomes for the fetus^13–18^.

A multicenter study in the US found that neonates born to mothers with preeclampsia or GHT are 2.9-fold more likely to receive intensive care than those born to normotensive mothers^19^. Women with unmanaged GDM are also at an elevated risk of developing complications during pregnancy, delivery, and the postpartum period^20–24^. Poor glycemic control increases adverse infant outcomes as well^25–32^. Pregnant women may also experience both HDP and GDM^33–36^. Women with HDP or those with both HDP and GDM are at elevated risk for subsequent hypertension and DM after delivery^33^.

However, previous studies have rarely investigated the complications and adverse birth outcomes associated with co-existing GDM and HDP. Most studies have evaluated pregnant women with only one of these disorders. The presence of both disorders during pregnancy may pose a greater health impact on mothers and infants.

In this study, we used large insurance claims data to investigate risks of adverse obstetric and neonatal outcomes in pregnant women with HDP alone and with both HDP and GDM. We compared one obstetric and 11 adverse neonatal outcomes in these two groups of women, comparing to reference women without HDP and GDM. We further assessed whether preeclampsia or eclampsia during pregnancy contributed to adverse outcomes.

## Results

### Demographics characteristics of study groups

**Figure 1:**
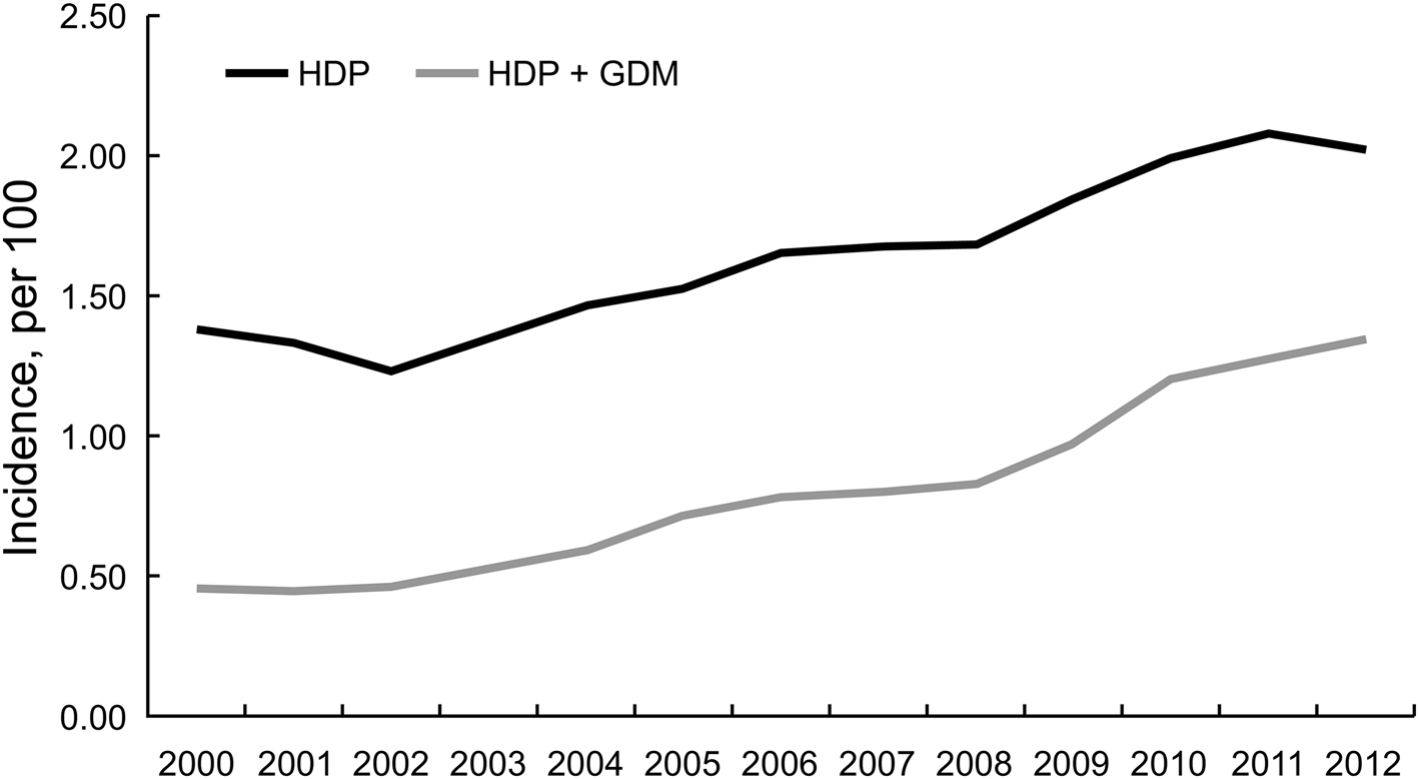
Twelve-year trend of incidence (per 100) of hypertensive disorders of pregnancy (HDP) and gestational diabetes mellitus (GDM) in pregnancy women.

Age distributions were similar among the three study groups, with a mean age of approximately 33 years; 36.4% of women were 30–34 years old (Table 1). The comparison group had slightly less rural residents, but had higher white-collar employees. Baseline prevalence rates of comorbidities in the three study groups were all less than 0.5%. Figure 1 shows the increasing trends of annual incidence (per 100) in pregnant women with HDP and with both HDP/GDM. The annual incidence rates of HDP were higher than that of HDP/GDM and increased considerably from 2000 to 2012.

**Table 1:** Demographics and comorbidities in women with hypertension during pregnancy (HDP), women with HDP and gestational diabetes mellitus (GDM), and comparison group.

	Comparison N = 19,442		HDP N = 7775		HDP/GDM N = 1946		p-value
	N	%	N	%	N	%	
**Age, years**							0.99
16–29	4890	25.2	1956	25.2	489	25.1	
30–34	7080	36.4	2832	36.4	708	36.4	
35–39	5800	29.8	2320	29.8	580	29.8	
40–45	1672	8.6	667	8.6	169	8.7	
Mean ± SD^a^	33.4 ± 4.95		33.3 ± 4.96		33.4 ± 4.98		0.96
**Urbanization level**							0.004
1 (highest)	6566	33.8	2482	31.9	673	34.6	
2	5670	29.2	2300	29.6	566	29.1	
3	3331	17.1	1349	17.4	315	16.2	
4	2313	11.9	919	11.8	211	10.8	
5 (lowest)	1562	8.0	725	9.3	181	9.3	
**Occupation**							0.11
White collar	12,086	62.2	4700	60.5	1200	61.7	
Blue collar	5229	26.9	2170	27.9	536	27.5	
Other	2127	10.9	905	11.6	210	10.8	
**Comorbidity**^**b**^							
Stroke	18	0.09	8	0.10	1	0.05	0.90
Ischemic heart disease	16	0.08	7	0.09	1	0.05	0.94
Heart failure	5	0.03	2	0.03	2	0.10	0.19
Renal disease	37	0.19	35	0.45	4	0.21	0.001
Obesity	5	0.03	14	0.18	7	0.36	0.001
Placental abruption^c^	75	0.39	37	0.48	4	0.21	0.23

Chi-square test used for categorical variables.

^a^ANOVA test.

^b^Baseline comorbidity using Fisher’s exact test.

^c^Complication of present pregnancy.

### Obstetric and neonatal adverse outcomes

Table 2 shows that HDP/GDM and HDP groups were approximately 1.7-fold more likely than the comparisons to deliver infants using Cesarean section (C-section) (66.0% and 66.7% versus 37.9%, respectively). Rates of adverse neonatal outcomes were all higher in the HDP group than in the comparison group, and increased further in the HDP/GDM group except respiratory distress syndrome (RDS). The adjusted odds ratios (aORs) of preterm delivery were 3.92 (95% CI 3.64–4.21) and 4.84 (95% CI 4.34–5.40), respectively, for women with HDP and with HDP/GDM relative to comparisons. The rates of neonatal hypoglycemia were more than fivefold greater in the HDP group and HDP/GDM group than in the comparisons with aORs of 5.20 (95% CI 3.91–6.92) and 8.68 (95% CI 6.13–12.3), respectively. The rate of large gestation age (LGA) was much greater in the HDP/GDM group than in both the comparisons and the HDP group, with aORs of 31.7 (95% CI 16.5–60.9) relative to the comparisons and 19.4 (95% CI 9.01–41.9) compared to the HDP group.

**Table 2:** Adjusted odds ratio (aOR) and 95% confidence interval (CI) of pregnancy outcomes in HDP women with and without GDM.

Outcome	Comparison N = 19,442		HDP N = 7775		HDP vs comparison	HDP/GDM N = 1946		HDP/GDM vs comparison	HDP/GDM vs HDP
	n	%	n	%^a^	aOR (95% CI)	n	%^a^	aOR (95% CI)	aOR (95% CI)
Cesarean section	7369	37.9	5185	66.7	3.32 (3.14–3.51)***	1285	66.0	3.22 (2.92–3.55)***	0.97 (0.85–1.08)
Preterm delivery	1613	8.30	2036	26.2	3.92 (3.64–4.21)***	592	30.4	4.84 (4.34–5.40)***	1.24 (1.11–1.38)***
SGA (low birth weight)	416	2.14	880	11.3	5.81 (5.15–6.55)***	244	12.5	6.57 (5.56–7.75)***	1.13 (0.97–1.32)
LGA (large baby)	12	0.06	8	0.10	1.65 (0.67–4.05)	37	1.90	31.7 (16.5–60.9)***	19.4 (9.01–41.9)***
Birth trauma	71	0.37	44	0.57	1.55 (1.06–2.26)*	28	1.44	3.99 (2.57–6.20)***	2.59 (1.61–4.18)***
Stillbirth	0	0.00	0	0.00	NA	0	0.00	NA	NA
PDA	67	0.34	89	1.14	3.34 (2.43–4.59)***	43	2.21	6.56 (4.46–9.65)***	1.98 (1.37–2.85)***
PFO (ASD)	99	0.51	90	1.16	2.26 (1.70–3.01)***	45	2.31	4.61 (3.22–6.58)***	2.05 (1.43–2.94)***
VSD	38	0.20	16	0.21	1.05 (0.59–1.89)	7	0.36	1.85 (0.83–4.15)	1.78 (0.73–4.32)
Jaundice	1682	8.65	1190	15.3	1.90 (1.76–2.06)***	426	21.9	2.95 (2.62–3.32)***	1.56 (1.37–1.76)***
RDS	126	0.65	304	3.91	6.18 (5.01–7.62)***	71	3.65	5.77 (4.30–7.75)***	0.93 (0.72–1.22)
Neonatal hypoglycemia	71	0.37	145	1.86	5.20 (3.91–6.92)***	60	3.08	8.68 (6.13–12.3)***	1.67 (1.23–2.27)***

HDP: hypertension during pregnancy without gestational diabetes mellitus; GDM: gestational diabetes mellitus; aOR: adjusted odds ratio controlling for demographic factors and baseline comorbidities; PDA, patent ductus arteriosus; PFO, foramen ovale/atrial septal defect; VSD, ventricular septal defect; RDS, respiratory distress syndrome.

*p < 0.05, **p < 0.01, ***p < 0.001.

^a^The sum over 100% due to cases with more than one outcome.

### Outcomes associated with preeclampsia and eclampsia

Table 3 shows that the C-section delivery rate increased steadily with hypertension status to the highest of 80.5% in women with eclampsia. The preterm delivery rate was much greater in women with preeclampsia or eclampsia than in women with only GHT and comparisons (30.6% or 29.0% versus 19.8% and 8.30%, respectively). Eclampsia or preeclampsia also led to higher risks of preterm delivery, small gestation age (SGA), patent ductus arteriosus (PDA), patent foramen oval PFO (ASD), RDS and neonatal hypoglycemia. Large differences existed for SGA rates with aORs of 10.8 (95% CI 7.42–15.8), 7.36 (95% CI 6.46–8.38) and 3.59 (95% CI 3.04–4.24) associated with eclampsia, preeclampsia and GHT, respectively.

**Table 3:** Comparison in obstetric and adverse neonatal outcomes associated with preeclampsia/eclampsia in women with hypertension during pregnancy (HDP) with and without gestational diabetes mellitus (GDM).

	Cesarean section				Preterm delivery			SGA		
	Total N	n	%	aOR (95% CI)	n	%	aOR (95% CI)	n	%	aOR (95% CI)
Comparison	19,442	7369	37.9	1.00	1613	8.30	1.00	416	2.14	1.00
**HDP/non-GDM**										
Only GHT	3170	1800	56.8	2.15 (1.99–2.32)***	629	19.8	2.72 (2.46–3.01)***	234	7.38	3.59 (3.04–4.24)***
Preeclampsia	4415	3232	73.2	4.58 (4.26–4.93)***	1352	30.6	4.89 (4.05–5.31)***	610	13.8	7.36 (6.46–8.38)***
Eclampsia	190	153	80.5	7.21 (5.02–10.4)***	55	29.0	4.57 (3.32–6.29)***	36	19.0	10.8 (7.42–15.8)***
**HDP/GDM**										
Only GHT/GDM	1077	665	61.8	2.63 (2.32–2.99)***	302	28.0	4.29 (3.72–4.95)***	111	10.3	5.22 (4.19–6.50)***
Preeclampsia/GDM	851	606	71.2	4.17 (3.58–4.85)***	284	33.4	5.57 (4.79–6.49)***	131	15.4	8.42 (6.82–10.4)***
Eclampsia/GDM	18	14	77.8	6.75 (2.20–20.7)***	6	33.3	6.02 (2.25–16.1)***	2	11.1	6.41 (1.46–28.1)*

	LGA				Birth trauma			Stillbirth		
Comparison	19,442	12	0.06	1.00	71	0.37	1.00	0	0.00	1.00
**HDP/non-GDM**										
Only GHT	3170	5	0.16	2.59 (0.91–7.37)	20	0.63	1.72 (1.05–2.83)*	0	0.00	NA
Preeclampsia	4415	3	0.07	1.08 (0.30–3.83)	24	0.54	1.50 (0.94–2.38)	0	0.00	NA
Eclampsia	190	0	0.00	NA	0	0.00	NA	0	0.00	NA
**HDP/GDM**										
Only GHT/GDM	1077	14	1.30	21.9 (10.1–47.6)***	19	1.76	4.89 (2.94–8.15)***	0	0.00	NA
Preeclampsia/GDM	851	23	2.70	44.5 (22.0–89.9)***	9	1.06	2.94 (1.46–5.91)**	0	0.00	NA
Eclampsia/GDM	18	0	0.00	NA	0	0.00	NA	0	0.00	NA

	PDA				PFO (ASD)			VSD		
Comparison	19,442	67	0.34	1.00	99	0.51	1.00	38	0.20	1.00
**HDP/non-GDM**										
Only GHT	3170	21	0.66	1.91 (1.17–3.13)**	28	0.88	1.70 (1.11–2.59)*	11	0.35	1.73 (0.89–3.40)
Preeclampsia	4415	65	1.47	4.33 (3.07–6.10)***	62	1.40	2.78 (2.02–3.83)***	5	0.11	0.59 (0.23–1.49)
Eclampsia	190	3	1.58	4.64 (1.45–14.9)**	0	0.00	NA	0	0.00	NA
**HDP/GDM**										
Only GHT/GDM	1077	17	1.58	4.64 (2.72–7.93)***	20	1.86	3.64 (2.24–5.92)***	3	0.28	1.41 (0.43–4.57)
Preeclampsia/GDM	851	26	3.06	9.19 (5.81–14.5)***	24	2.82	5.70 (3.63–8.96)***	4	0.47	2.47 (0.88–6.94)
Eclampsia/GDM	18	0	0.00	NA	1	5.56	13.0 (1.70–98.9)*	0	0.00	NA

	Jaundice				RDS			Neonatal hypoglycemia		
Comparison	19,442	1682	8.65	1.00	126	0.65	1.00	71	0.37	1.00
**HDP/non-GDM**										
Only GHT	3170	490	15.5	1.91 (1.72–2.13)***	69	2.18	3.33 (2.48–4.48)***	46	1.45	3.97 (2.73–5.76)***
Preeclampsia	4415	671	15.2	1.89 (1.72–2.09)***	218	4.94	7.98 (6.39–9.96)***	98	2.22	6.27 (4.61–8.53)***
Eclampsia	190	29	15.3	1.94 (1.30–2.89)**	17	8.95	15.0 (8.84–25.5)***	1	0.53	1.54 (0.21–11.1)
**HDP/GDM**										
Only GHT/GDM	1077	222	20.6	2.71 (2.32–3.17)***	32	2.97	4.61 (3.11–6.84)***	29	2.69	7.45 (4.81–11.5)***
Preeclampsia/GDM	851	201	23.6	3.28 (2.78–3.87)***	39	4.58	7.42 (5.14–10.7)***	31	3.64	10.5 (6.82–16.1)***
Eclampsia/GDM	18	3	16.7	2.33 (0.67–8.08)*	0	0.00	NA	0	0.00	NA

aOR: adjusted odds ratio after controlling for age, urbanization level, renal disease and obseity; HDP: hypertension during pregnancy; GHT: gestational hypertension; non-GDM: no gestational diabetes mellitus. SGA, small gestation age; LGA, large gestation age; PDA, patent ductus arteriosus; PFO, foramen ovale/atrial septal defect; VSD, ventricular septal defect; RDS, respiratory distress syndrome.

*p < 0.05, **p < 0.01, ***p < 0.001.

Table 3 also shows that all rates of neonatal adverse outcomes were greater in the HDP/GDM group than in the HDP group. Most these adverse outcomes in HDP/GDM women with eclampsia or preeclampsia also increased further, to levels greater than those in HDP women with eclampsia or preeclampsia. The risk of delivering a LGA baby was particularly higher in HDP/GDM women (aOR = 21.9, 95% CI 10.1–47.6) and in those with preeclampsia (aOR = 44.5, 95% CI 22.0–89.9).

## Discussion

It is well known that pregnant women with HDP or GDM are at elevated risks of subsequent adverse maternal and neonatal health conditions. Our study showed that HDP is associated with increased C-section delivery with higher preterm delivery and 9 adverse neonatal outcomes than comparisons without HDP. The incidence of HDP in our study was 2.41% (65,021/2,694,351). The recent Canadian statistics showed that hypertension affected approximately 7.0% of pregnant women^3^. Another recent study analyzed the national data of China and found a HDP rate of 3.89% (n = 270,982) among 6,970,032 pregnancies^13^. Our study revealed that 4.555% of women had a concomitant diagnosis of GDM and HDP. The presence of both HDP and GDM during pregnancy posed greater risks of adverse neonatal outcomes than the presence of HDP alone. The risks of most adverse outcomes increased to more higher levels in women with preeclampsia or eclampsia developed in the HDP group and HDP/GDM group.

### Comparing neonatal outcomes between HDP and HDP/GDM groups

The effect of HDP in pregnant women varies among populations. A recent cross-sectional study based on 3,659,553 women with a live birth delivery among the US states found HDP affecting 4.3% to 9.3% pregnancies^37^. The risk of developing HDP in our study population might not higher than other population^1–3,37^. Our study found the preterm delivery in women with HDP was more than threefold higher than comparisons. An earlier US study showed that the adjusted relative risk of preterm delivery in women with HDP was 1.87 compared to references^38^. In our study, the highest incidence among other adverse neonatal outcomes in HDP women was jaundice, followed by SGA and RDS, with few cases of LGA. However, the estimated relative risk was the highest for RDS with an aOR of 6.18. The risk is higher than the finding in a US nest case–control study within the Calcium for Preeclampsia Prevention trial, with an aOR of 2.18 for RDS associated with HDP^39^. For premature infants, the RDS is a common cause of respiratory failure. This is due to insufficient production of pulmonary surfactant and the immature structure of the lung^40^. Previous studies found the impact of preeclampsia on RDS conflicting^41–45^. However, we are unable to conclude the impact of HTN on RDS because there is no data on antenatal corticosteroid use.

We found that children born to women with HDP/GDM had greater incidence adverse neonatal outcomes than children born to women with HDP, except RDS. Pregnant women with GDM are known at a higher risk of having newborns of LGA. A Swedish cohort study found an OR of 3.43 (95% CI 3.21–3.67) for LGA associated with GDM based on the birth registry data of 1,260,297 women^27^. This study did not evaluate LGA and SGA for women with both HDP and GDM. We note in our study that there were more neonatal SGA than LGA (12.5% versus 1.90%) born to women with HDP/GDM. However, the aOR of LGA was much greater than that of SGA (31.7 versus 6.57) relative to the comparison group. The corresponding aORs reduced to 21.9 and 5.22 for infants born to women with GHT/GDM. It seems hypertension may interact with diabetes exerting increased risk of adverse neonatal outcomes in pregnant women.

### Preeclampsia or eclampsia impact

Our data also showed that most other investigated adverse neonatal outcomes increased further, in addition to the risk of preterm delivery and low birth weight, in women with preeclampsia or eclampsia; the impacts were even greater in the HDP/GDP group than in the HDP group.

Our data show that large portions of women in both the HDP group and HDP/GDM group developed preeclampsia (56.8% versus 43.7%, or 4415/7775 versus 851/1946), but 2.44% (n = 190) and 0.92% (n = 18) developed eclampsia, respectively. Infants born to pregnant women in the HDP group with preeclampsia or eclampsia developed had the highest SGA rates, with very low rate of LGA. Whereas infants born to the HDP/GDM group with preeclampsia were at the highest risk of LGA. The OR of giving birth to a LGA baby increased further to 44.5 in women with preeclampsia in the HDP/GDM group. This is an exceptional finding has not been reported previously^22,42,44,45^. A recent study evaluating 30,139 pregnancies in Ontario, Canada, also associated pre-pregnancy diabetes with increased risk of LGA with an adjusted relative risk of 28.9 in preterm births^46^. However, the absolute rate of LGA was smaller in our study than in the Ontario study (2.7% versus 6.4%).

### Congenital malformations are associated with preterm delivery

Studies have associated GDM and hypertensive disorders with congenital defects, including congenital heart defects^47–50^, particularly in women with preterm preeclampsia^51–53^. A meta-analysis based on 15 cohort studies found a relative risk of 1.16 (1.07–1.25) for major congenital malformations in the offspring of women with GDM^47^. A Chinese study found that gestational diabetes is one of risk factors associated with the development of congenital heart disease based on the data of 90,796 infants^48^. A Demark study with 1,972,857 singleton pregnancies found a greater risk of offspring congenital heart defects in women with early preterm preeclampsia than in women with late preterm preeclampsia (OR 7.00 versus 2.82)^52^. Our study found elevated risks of PDA and PFO in both HDP group and HDP/GDM group, and increased further in women with Preeclampsia/GDM. However, we are unable to assess whether these neonatal abnormalities diagnosed were congenital defects without further follow up evaluation for these children. PFO and ASD are likely linked to prematurity. But, we found that VSD was the only adverse birth outcome of congenital defect presented in the 3 study groups ranging from 0.20 to 0.36%, which were not different between the groups.

Our study also found a high risk of neonatal hypoglycemia with an aOR of 10.5, in women with preeclampsia/GDM relative to comparisons. The development of neonatal hypoglycemia might be influenced by the early gestational age at delivery. Managing GDM by tight glycemic control during pregnancy is essential to effectively reduce the abnormalities. A secondary analysis from the North American Hyperglycemia and Adverse Pregnancy Outcome Study also found that women with GDM were at a 2.11-fold higher risk of neonatal hypoglycemia than those without GDM^54^. The development of neonatal hypoglycemia might be influenced by the early gestational age at delivery. Managing GDM by tight glycemic control during pregnancy is essential to effectively reduce the abnormalities.

## Strengths and limitations

To the best of our knowledge, this is the first Asian study to evaluate multiple birth outcomes in pregnant women with HDP and HDP/GDM and in those who developed preeclampsia or eclampsia. Although this study was strengthened by the use of the large insurance claims database, there were several limitations. First, information on body mass index, lifestyle of drinking, smoking and diet, and family health history was unavailable to adjust for these potential confounders in data analyses. However, the impact from some of these factors might be minor because pregnant women are more likely to avoid unhealthy behaviors. Smoking and drinking are rare habits in women in Taiwan and obesity is not prevalent as well. The study results might not be generalizable to non-Chinese populations and populations with higher rates of obesity. Second, the HDP and HDP/GDM cohorts utilized more medical interventions, which might increase the diagnosis of adverse outcomes in these groups. Third, information on the severity of disorders during pregnancy was unavailable for analyses. Misclassification would tend to increase the observed magnitude associated with severe conditions. However, it is unlikely that we have misclassified women with preeclampsia or eclampsia, as these conditions made a strong impact that had not been previously reported. Fourth, we established groups of women with HDP and women with both HDP and GDM, without a group of GDM. Therefore, the impact associated with GDM alone or with the severity of GDM could not be evaluated in this study, but we were able to subdivide hypertensive disorders of pregnancy into pregnancy-induced hypertension and preeclampsia or eclampsia. Fifth, a high incidence of preterm delivery was observed in this study. However, we were unable to identify the spontaneous preterm delivery to further evaluate the attribution to the neonatal outcomes.

## Conclusions

This study showed a steady increase in risks of adverse neonatal outcomes for pregnant women with HDP, HDP/GDM and those with preeclampsia or eclampsia. Because of the progressive nature of HDP and GDM, early delivery is usually recommended to minimize the maternal morbidity and mortality, especially for the more severe presentations of HDP or GDM, such as preeclampsia and eclampsia.

Our findings underscore the need for prenatal care with careful attention to pregnant women with HDP, particularly to women with both HDP and GDM. Obstetricians may need to screen for fetal abnormalities in pregnant women with these disorders, particularly in those with preeclampsia or eclampsia. It is important to detect and treat HDP and GDM early to reduce obstetrical complications and adverse neonatal outcomes, tight glycemic control and hypertension control are prudent. Future studies need to evaluate the risks and benefits of labor and Cesarean delivery for women with HDP and/or GDM.

## Methods

### Data sources

The Department of Health Insurance in Taiwan is a government-managed system established in 1995 through integration of 11 public insurance programs to create a universal insurance system, which is compulsory for all residents. Approximately 99% of the 23 million Taiwanese people have been covered in the program since 1997. The National Health Research Institutes (NHRI) of Taiwan established several data files of reimbursement claims available for research at the inception of 1997. For this study, we aimed to investigate the neonatal outcomes for women with a singleton pregnancy at their first birth. To minimize the inclusion of multiple pregnancies and multiple births, we used the whole population claims data for the period of 2000–2012.

To ensure the privacy of the participants, all the data were linked with surrogate identifications processed by NHRI before releasing to researchers. Information on patient demographic status and health care received were available. Diseases and other health care events were coded using the International Classification of Diseases, Ninth Revision, Clinical Modification (ICD-9-CM). The use of insurance claim data was approved by the Research Ethics Committee of China Medical University and Hospital, Taichung, Taiwan (CMUH104-REC2-115). We adhered to the principles in the Declaration of Helsinki in this study. Informed consent of patients was not required due to the retrospective design of the study and the use of scrambled data.

### Study population

**Figure 2:**
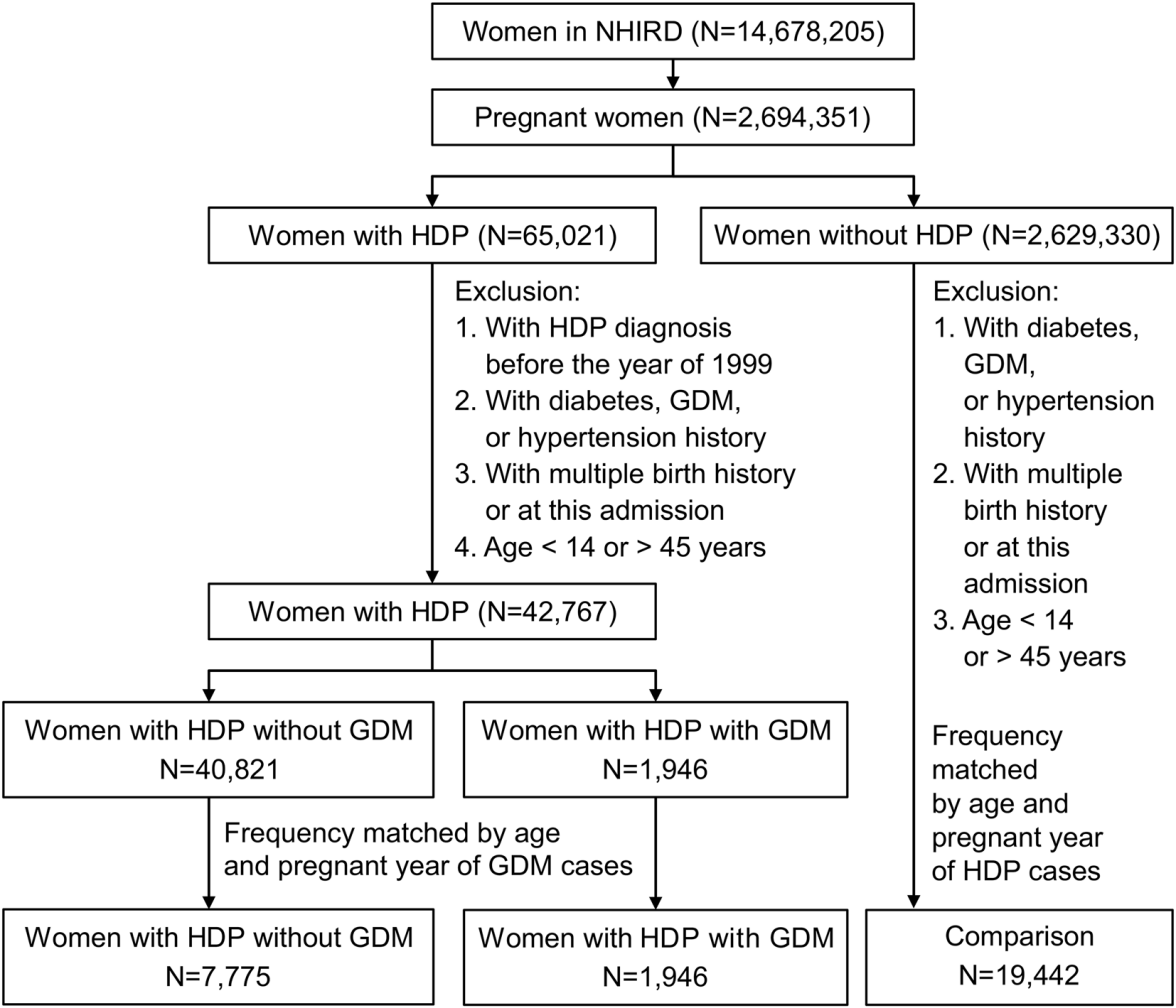
Flow chart for establishing study cohorts.

From the claims data of the whole female insured population (N = 14,678,205), we identified 2,694,351 women with pregnancy diagnosed from 2000 to 2012 in Taiwan (Fig. 2). Of these women 65,021 women had HDP (ICD-9-CM 642). The date of HDP diagnosis was defined as the index date. We exclude those with HDP diagnosed before the year of 2000; those with a history of diabetes, GDM and hypertension history (ICD-9-CM 250, 648.0, 648.8 and 401–405 respectively); and those younger than 14 or older than 45 years; and those with multiple birth history (ICD-9-CM 651, 652.6 and 761.5) or at this admission. Women with multifetal gestations were also excluded. Of the remaining 42,767 women with HDP were eligible for this study. Among them, 1946 women who had also developed GDM during pregnancy were identified as the HDP/GDM cohort. Among women with only HDP, we selected a cohort with a size fourfold (N = 7775) of the HDP/GDM cohort, frequency matched by age and the diagnosis year of HDP. From 2,629,330 pregnant women without HDP, we randomly selected a comparison cohort, with a size twofold of the combined size of HDP cohort and HDP/GDM cohort, frequency matched by age and pregnant year. The exclusion criteria used for selecting the HDP/GDM and HDP cohorts were applied for establishing the comparison cohort, with a sample size of 19,442 women.

### Birth outcomes and associated factors

For each patient, we examined normal delivery (650) and Cesarean section (ICD-9 code operation 74), and 11 adverse neonatal outcomes from the birth records, including preterm delivery (ICD-9 code: 644 and 765.1), low birthweight (small for gestational age (SGA) (ICD-9 code: 765), large baby (large for gestational age (LGA)) (ICD-9 code: 766), birth trauma (ICD-9 code: 767), stillbirth (ICD-9 code: 768.0, 768.1), patent ductus arteriosus (PDA, ICD-9 code: 747.0), patent foramen ovale/atrial septal defect (PFO [ASD], ICD-9 code: 745.5), ventricular septal defect (VSD, ICD-9 code: 745.4), jaundice (ICD-9 code: 774), respiratory distress syndrome (RDS, ICD-9 code: 769), and neonatal hypoglycemia (ICD-9 code: 775.6). The demographic data file provided information on age (16–29, 30–34, 35–39, and 40–45 years), urbanization level, and occupation (white-collar, blue-collar, and others). We categorized all residential areas into five urbanization levels from the highest urbanized level as 1 to the lowest level as 5. We also searched for comorbidities that were potentially linked to obstetric birth outcomes including stroke (ICD-9-CM 430–438), heart failure (ICD-9-CM 428), ischemic heart disease (ICD-9-CM 410–414), renal disease (ICD-9-CM 580–589), placental abruption (ICD-9-CM 641.2), and obesity (ICD-9-CM 278, 783.1). All baseline comorbidities were defined before the index date.

### Statistical analyses

We used SAS software version 9.4 (SAS Institute Inc., Cary, NC) to perform the data analysis for this study, with a p-value of < 0.05 considered statistically significant. The Chi-square test and Fisher's exact test were used to examine differences of categorical variables between HDP, HDP/GDM and comparison groups, including age, urbanization level and occupation, and comorbidities. Analysis of variance (ANOVA) was used to examine differences of mean ages among the three groups. Multivariable logistic regression was used to estimate the adjusted odds ratio (aOR) with 95% confidence interval (CI) of each birth outcome measured for the HDP group and the HDP/GDM group, relative to the comparison group. The aOR was estimated after controlling for age, urbanization level and occupation, and significant comorbidities at the baseline. The aOR of each birth outcome was also measured for women with HDP/GDM compared to women with HDP. We further calculated the aOR of each birth event associated with GHT, preeclampsia and eclampsia (ICD-9-CM 642.4–642.6) in the HDP group, and associated with GHT/GDM, preeclampsia and eclampsia in the HDP/GDM group, controlling for age, urbanization level, occupation, and comorbidity.

## Supplementary Information

Supplementary Information.

## Data Availability

The data that support the findings of this study were obtained from National Health Insurance Research database (NHIRD) of the Ministry of Health and Welfare, established by the National Health Research Institutes of Taiwan. The Ministry of Health and Welfare approved our use of the data. Any researcher interested in accessing this dataset can submit an application to the Ministry of Health and Welfare requesting access. We are not eligible to duplicate and disseminate the database. For further access to the database, please contact the Ministry of Health and Welfare (Email: stcarolwu@mohw.gov.tw) for further assistance. Taiwan Ministry of Health and Welfare Address: 488 Zhongxiao E. Rd. Sec. 6, Nangang Dist., Taipei 115, Taiwan (R.O.C.). Phone: + 886-2-8590-6848).
