# Supplementary material for: Population-based study on birth outcomes among women with hypertensive disorders of pregnancy and gestational diabetes mellitus
Source: Sci Rep. 2021 Aug 30;11:17391. doi: 10.1038/s41598-021-96345-0 (PMC8405617; doi:10.1038/s41598-021-96345-0)
Supplement: Supplementary file 1 — Supplementary Information. [file 41598_2021_96345_MOESM1_ESM.docx]

**Supplement Table**

**Supplement Table 1**. Odds ratio (OR) and 95% confidence intervals (CIs) of **Maternal outcomes** in HDP* women with and without GDM^†^

|  | **Comparison**  **N=19442** | | **HDP**  **N=7775** | | **HDP vs comparison** | **HDP/GDM**  **N=1946** | | **HDP/GDM vs comparison** | **HDP/GDM vs HDP** |
| --- | --- | --- | --- | --- | --- | --- | --- | --- | --- |
| Outcome | N | % | N | %^a^ | aOR^‡^ (95% CI) | N | %^a^ | aOR^‡^ (95% CI) | aOR ^‡^ (95% CI) |
| Edema or excessive weight gain | 0 | 0.00 | 15 | 0.19 | NA | 10 | 0.51 | NA | 2.64 (1.18-5.91) |
| Seizure | 5 | 0.03 | 13 | 0.17 | 6.00 (2.13-16.9) | 2 | 0.10 | 3.96 (0.77-20.4) | 0.66 (0.15-2.96) |
| DIC | 8 | 0.04 | 21 | 0.27 | 6.68 (2.96-15.1) | 3 | 0.15 | 3.76 (0.99-14.2) | 0.56 (0.17-1.89) |
| Embolism | 6 | 0.03 | 4 | 0.05 | 1.74 (0.49-6.17) | 1 | 0.05 | 1.66 (0.20-13.8) | 0.94 (0.11-8.43) |
| APH | 76 | 0.39 | 25 | 0.32 | 0.83 (0.53-1.30) | 5 | 0.26 | 0.66 (0.27-1.64) | 0.80 (0.30-2.09) |
| Abortion | 70 | 0.36 | 14 | 0.18 | 0.50 (0.28-0.88) | 4 | 0.21 | 0.58 (0.21-1.58) | 1.22 (0.40-3.72) |
| Stroke | 4 | 0.02 | 6 | 0.08 | 3.80 (1.07-13.5) | 1 | 0.05 | 2.58 (0.29-23.1) | 0.67 (0.08-5.58) |
| PPH | 191 | 0.98 | 159 | 2.05 | 2.10 (1.70-2.60) | 38 | 1.95 | 2.00 (1.41-2.84) | 0.95 (0.67-1.36) |
| Death | 28 | 0.14 | 36 | 0.46 | 3.22 (1.96-5.28) | 9 | 0.46 | 3.22 (1.52-6.83) | 0.99 (0.48-2.05) |

*HDP: hypertension during pregnancy without gestational diabetes mellitus; ^†^GDM: gestational diabetes mellitus; ^‡^aOR: adjusted odds ratio after controlling for age, urbanization level, renal disease, and obesity.

DIC, Disseminated Intravascular Coagulation ; APH, Antepartum Hemorrhage ; PPH, Postpartum Hemorrhage.

^a^The sum may be more than 100% due to some cases have more than one outcome

**Supplement Table 2.** Adverse **maternal outcomes** associated with preeclampsia/eclampsia in women with hypertension during pregnancy (HDP^†^) without gestational diabetes mellitus (GDM)

|  | **Edema or excessive weight gain** | | | | **Seizure** | | | **DIC** | | |
| --- | --- | --- | --- | --- | --- | --- | --- | --- | --- | --- |
|  | **Total N** | **n** | **%** | **aOR^*^ (95% CI)** | **n** | **%** | **aOR^*^ (95% CI)** | **n** | **%** | **aOR^*^ (95% CI)** |
| Comparison | 19442 | 0 | 0.00 | 1.00 | 5 | 0.03 | 1.00 | 8 | 0.04 | 1.00 |
| HDP^†^/Non-GDM |  |  |  |  |  |  |  |  |  |  |
| Only GHT^‡^ | 3170 | 7 | 0.22 | NA | 1 | 0.03 | 1.20 (0.14-1.02) | 3 | 0.09 | 2.38 (0.63-8.96) |
| Preeclampsia/eclampsia | 4605 | 8 | 0.17 | NA | 12 | 0.26 | 9.60 (3.36-27.4) | 18 | 0.39 | 9.68 (4.21-22.3) |
|  | **Embolism** | | | | **APH** | | | **Abortion** | | |
| Comparison | 19442 | 6 | 0.03 | 1.00 | 76 | 0.39 | 1.00 | 70 | 0.36 | 1.00 |
| HDP^†^/Non-GDM |  |  |  |  |  |  |  |  |  |  |
| Only GHT^‡^ | 3170 | 1 | 0.03 | 1.11 (0.13-9.25) | 15 | 0.47 | 1.19 (0.68-2.08) | 10 | 0.32 | 0.87 (0.45-1.68) |
| Preeclampsia/eclampsia | 4605 | 3 | 0.07 | 2.17 (0.54-8.67) | 10 | 0.22 | 0.56 (0.29-1.09) | 4 | 0.09 | 0.24 (0.09-0.66) |
|  | **Stroke** | | | | **PPH** | | | **Death** | | |
| Comparison | 19442 | 4 | 0.02 | 1.00 | 191 | 0.98 | 1.00 | 28 | 0.14 | 1.00 |
| HDP^†^/Non-GDM |  |  |  |  |  |  |  |  |  |  |
| Only GHT^‡^ | 3170 | 1 | 0.03 | 1.53 (0.17-13.7) | 79 | 2.49 | 2.55 (1.95-3.32) | 11 | 0.35 | 2.34 (1.16-4.71) |
| Preeclampsia/eclampsia | 4605 | 5 | 0.11 | 5.42 (1.45-20.2) | 80 | 1.74 | 1.80 (1.38-2.34) | 25 | 0.54 | 3.81 (2.22-6.54) |

*aOR: adjusted odds ratio after controlling for age, urbanization level, occupation, heart failure, and renal disease; ^†^HDP: hypertension during pregnancy; ^‡^GHT: gestational hypertension; non-GDM: no gestational diabetes mellitus.

DIC, Disseminated Intravascular Coagulation ; APH, Antepartum Hemorrhage ; PPH, Postpartum Hemorrhage.

**Supplement Table 3.** Adverse **maternal outcomes** associated with preeclampsia/eclampsia in women with both hypertension during pregnancy (HDP^†^) and gestational diabetes mellitus (GDM^‡^)

|  | **Edema or excessive weight gain** | | | | **Seizure** | | | **DIC** | | |
| --- | --- | --- | --- | --- | --- | --- | --- | --- | --- | --- |
|  | **Total N** | **n** | **%** | **aOR^*^ (95% CI)** | **n** | **%** | **aOR^*^ (95% CI)** | **n** | **%** | **aOR^*^ (95% CI)** |
| Comparison | 19442 | 0 | 0.00 | 1.00 | 5 | 0.03 | 1.00 | 8 | 0.04 | 1.00 |
| HDP^†^/Non-GDM |  |  |  |  |  |  |  |  |  |  |
| Only GHT^‡^ | 3170 | 5 | 0.46 | NA | 0 | 0.00 | NA | 2 | 0.19 | 4.63 (0.98-21.9) |
| Preeclampsia/eclampsia | 4605 | 5 | 0.58 | NA | 2 | 0.23 | 7.27 (1.33-39.8) | 1 | 0.12 | 2.74 (0.34-21.9) |
|  | **Embolism** | | | | **APH** | | | **Abortion** | | |
| Comparison | 19442 | 6 | 0.03 | 1.00 | 76 | 0.39 | 1.00 | 70 | 0.36 | 1.00 |
| HDP^†^/Non-GDM |  |  |  |  |  |  |  |  |  |  |
| Only GHT^‡^ | 3170 | 1 | 0.09 | 2.95 (0.35-24.5) | 5 | 0.46 | 1.18 (0.48-2.93) | 3 | 0.28 | 0.76 (0.24-2.42) |
| Preeclampsia/eclampsia | 4605 | 0 | 0.00 | NA | 0 | 0.00 | NA | 1 | 0.12 | 0.33 (0.05-2.37) |
|  | **Stroke** | | | | **PPH** | | | **Death** | | |
| Comparison | 19442 | 4 | 0.02 | 1.00 | 191 | 0.98 | 1.00 | 28 | 0.14 | 1.00 |
| HDP^†^/Non-GDM |  |  |  |  |  |  |  |  |  |  |
| Only GHT^‡^ | 3170 | 0 | 0.00 | NA | 24 | 2.23 | 2.25 (1.46-3.45) | 7 | 0.65 | 4.54 (1.98-10.4) |
| Preeclampsia/eclampsia | 4605 | 1 | 0.12 | 5.86 (0.65-52.6) | 14 | 1.61 | 1.68 (0.97-2.91) | 2 | 0.23 | 1.62 (0.39-6.82) |

*aOR: adjusted odds ratio after controlling for age, urbanization level, occupation, heart failure, and renal disease; ^†^HDP: hypertension during pregnancy; ^‡^GHT: gestational hypertension; non-GDM: no gestational diabetes mellitus.

DIC, Disseminated Intravascular Coagulation ;APH, Antepartum Hemorrhage ; PPH, Postpartum Hemorrhage;
